# Effects of Vanadium on the Structural and Optical Properties of Borate Glasses Containing Er^3+^ and Silver Nanoparticles

**DOI:** 10.3390/ma14133710

**Published:** 2021-07-02

**Authors:** Nur Adyani Zaini, Syafawati Nadiah Mohamed, Zakiah Mohamed

**Affiliations:** Faculty of Applied Sciences, Universiti Teknologi MARA, UiTM, Shah Alam 40450, Selangor, Malaysia; adyanizaini@gmail.com (N.A.Z.); syafawati@uitm.edu.my (S.N.M.)

**Keywords:** borate glasses, erbium-vanadium doped glasses, silver nanoparticles, UV–Vis spectroscopy, photoluminescence

## Abstract

The erbium-vanadium co-doped borate glasses, embedded with silver nanoparticles (Ag NPs), were prepared to improve their optical properties for potential optical fiber and glass laser application. The borate glasses with composition (59.5–*x*) B_2_O_3_–20Na_2_O–20CaO–*x*V_2_O_5_–Er_2_O_3_–0.5AgCl (*x* = 0–2.5 mol%) were successfully prepared by conventional melt-quenching method. The structural properties of glass samples were investigated by XRD, TEM and by Fourier transform infrared (FTIR) spectroscopy while optical properties were carried out by UV–Vis spectroscopy by measuring optical absorption and the emission properties were investigated by photoluminescence spectroscopy. The XRD patterns confirmed the amorphous nature of the prepared glass samples whilst the FTIR confirmed the presence of VO_4_, VO_5_, BO_3_ and BO_4_ vibrations. UV–Vis–NIR absorption spectra reveal eight bands which were located at 450, 490, 519, 540, 660, 780, 980, and 1550 nm corresponding to transition of ^4^F_5/2_, ^4^F_7/2_, ^2^H_11/2_, ^4^S_3/2_, ^4^F_9/2_, ^4^I_9/2_, ^4^I_11/2_, and ^4^I_13/2_, respectively. The optical band gap (E_opt_), Urbach energy and refractive index were observed to decrease, increase and increase, respectively, to the addition of vanadium. Under 800 nm excitation, three emission bands were observed at 516, 580 and 673 nm, which are represented by ^2^H_11/2_–^4^I_15/2_, ^4^S_3/2_–^4^I_15/2_ and ^4^F_15/2_–^4^I_15/2_, respectively. The excellent features of achieved results suggest that our findings may provide useful information toward the development of functional glasses.

## 1. Introduction

Boron-based oxides have unique properties, including high transparency, low melting point, good rare-earth ion solubility, low viscosity, high dielectric constant, low cost, large phonon energy (~1300–1500 cm^−1^) and easy preparation in bulk form. They also feature vibration resistance, low refractive index, low cation size and high bond strength [1,2,3,4,5,6]. These properties qualify boron-based glass as a suitable material for noble optical devices [1,2,3,4,5,6]. Boroxol is a form of pure boron B_2_O_3_. However, if B_2_O_3_ is added with some modifier oxides, BO_3_ (nonbridge oxide) units transform into BO_4_ (bridge oxide), which comprises some weakly attached BO_3_ triangles, BO_4_ tetrahedrons, some BO_4_^−^ units without the formation of nonbridge oxygen (NBO) [7,8], and a variety of superstructural units, such as tri-, penta-, tetra-, di-, pyro- and orthoborate. For example, when alkali or alkaline-earth metal oxide is added into glass as a modifier elastic, boron glass shows borate anomaly [5]. Such borate anomaly is explained by considering the transformation of three- to four-fold coordinated boron during the initial addition of a modifier oxide, but the high content of the modifier creates NBO [6].

Meanwhile, the incorporation of two dissimilar former glass produces a phenomenon called the mixed glass former effect (MGFE) [7]. When vanadium (V_2_O_5_) is introduced into boron (B_2_O_3_), borovanadate glass, which consists of a mixed network of the former, is formed. Vanadium is required in forming glass with the addition of other components through the conventional quenching method. However, the role of vanadium is dependent on its concentration [5]. At a high concentration, it can be considered as a former glass; at a low concentration, vanadium can be considered as a modifier [7,8]. The phase shifts due to vanadium have an influence on optical, structural, and electrical characteristics of borate glass [9,10]. The MGFE composition has attracted interest among researchers because of its intriguing structural and physical properties.

Vanadium is also used to overcome any clustering caused by high concentrations of erbium. A high concentration of erbium is normally required to achieve a strong emission [11], but a high doping level of erbium may cause clustering, which leads to luminescence quenching and large nonradiative losses [12]. Several approaches can be adopted to overcome this issue, and they include producing glass ceramic via heat treatment, introducing metallic nanoparticles (NPs) [11] and co-doping with various rare-earth ions or transition metals [13,14]. Previous reports confirmed that the emission intensity of system erbium ion-doped glass co-doped with other rare-earth ions, such as Tm^3+^, Nd^3+^ and Yb^3+^ is stronger than that of single erbium ion-doped glass. Thus, emission could be achieved by co-doping rare-earth ions with transition and metallic NPs.

The participation of vanadium ion in radiative transitions within the glass network has been studied in the emission spectra of 40Na_2_O–54SiO_2_–(5–*x*) ZrO_2_–Ho_2_O_3_–*x*V_2_O_5_ [13] and 40Na_2_O–54SiO_2_–(5–*x*) ZrO_2_–Sm_2_O_3_–*x*V_2_O_5_ glasses [15]. An additional band appears at 636 and 1095 nm because of the transition ^2^B_2_ → ^2^B_g_ and ^2^B_2_ → ^2^E_g_ when V_2_O_5_ is added. The addition of V_2_O_5_ in a host matrix has been suggested to improve luminescence efficiency and reduce phonon energies. Hence, the concentration of V_2_O_5_ is believed to be the local environment of rare-earth ions in the oxide glass and is not strictly dependent on the composition of the host matrix. Thus, the concentration of vanadium may contribute to different crystal field strengths. Several studies have explored the emission properties of V_2_O_5_-doped rare-earth glass, but further research is needed to facilitate a rich understanding of the role of V_2_O_5_ in glass modification.

In the current work, the effects of vanadium on the structural and optical properties of (59.5–*x*) B_2_O_3_–20Na_2_O–20CaO–*x*V_2_O_5_–Er_2_O_3_–0.5AgCl (x = 0, 0.5, 1.0, 1.5, 2.0 and 2.5 mol%) glasses were studied. The structural properties were examined by means of X-ray diffraction (XRD), Fourier transform infrared (FTIR) and transmission electron microscopy (TEM). Meanwhile, an ultraviolet–visible (UV–Vis) spectrometer and photoluminescence (PL) spectrometer were used to study the absorption and luminesce spectra of the glass samples that are required in the calculation of the Judd–Ofelt intensity parameter. Furthermore, the radiative properties, including the effective bandwidth, radiative transition probability, radiative lifetime, and branching ratio, were measured and analyzed.

## 2. Materials and Methods

### 2.1. Preparation of Glasses

Glass samples with the composition of (59.5–*x*) B_2_O_3_–20Na_2_O–20CaO–*x*V_2_O_5_–Er_2_O_3_–0.5AgCl (*x* = 0, 0.5, 1.0, 1.5, 2.0 and 2.5 mol%) were prepared through the conventional melt-quenching method. This composition enables the formation of transparent glass which is suitable for optical applications [7]. The appropriate amount of analytical-grade commercial powder boron oxide (B_2_O_3_), sodium carbonate (Na_2_CO_3_), calcium carbonate (CaCO_3_), vanadium oxide (V_2_O_5_), erbium oxide (Er_2_O_3_) and silver chloride (AgCl) (purity ≥99%) were mixed and weighed homogeneously. At 1150 °C, the homogeneous mixture was melted in alumina crucibles for 2 h. Then, the samples were quenched into a stainless plate and molded. Thereafter, the samples were annealed at 300 °C for 5 h in another furnace. After 5 h, the furnace automatically stopped the process and reduced the temperature gradually until room temperature was reached. The glass samples were then polished using sandpaper to obtain a parallel opposite surface with a thickness of approximately 5 mm for optical absorption and photoluminescent spectroscopy. The glass samples were powderized for XRD, TEM and infrared (IR) absorption characterization.

### 2.2. Characterization of Glasses

The XRD analysis of the glasses was conducted using X’Pert Pro Panalytical diffraction to confirm the amorphous property of the samples. The formation of a crystalline plane in the silver NPs was confirmed through TEM analysis. A small amount of powder samples was dispersed into acetone liquid by using an ultrasonic bath. The solution was then placed onto a copper grid to dry before it was ready for characterization. In determining the density of the glass samples, the Archimedes principle was applied [14]; here, the immersion medium was toluene (0.8669 g cm^−3^) [15,16] at room temperature. Meanwhile, the values of molar volume (*V_a_*) were calculated using the following equation:

(1) $$V_{a}=\frac{M_{V}}{\rho }$$

where $M_{V}$ is the molar mass of the samples.

A Perkin Elmer UV–Vis–NIR spectrophotometer in the range of 200–1000 nm was used to record absorption spectra of the glass samples. A Perkin Elmer model Spectrum One FTIR was used to investigate the IR absorption spectra of the glass samples; the functional group was within the range of 400–1600 cm^−1^. In the process, the powdered glass samples were mixed with KBr at a fixed ratio of 1:80. The mixture was then hand pressed into a pallet. The visible up-conversion emission measurement was performed in the wavelength region of 200–900 nm at room temperature by using the Perkin LS-55 luminesce spectrometer, in which a pulsed xenon lamp operated as the source of excitation.

## 3. Results

### 3.1. XRD, TEM and Physical Properties

**Figure 1:**
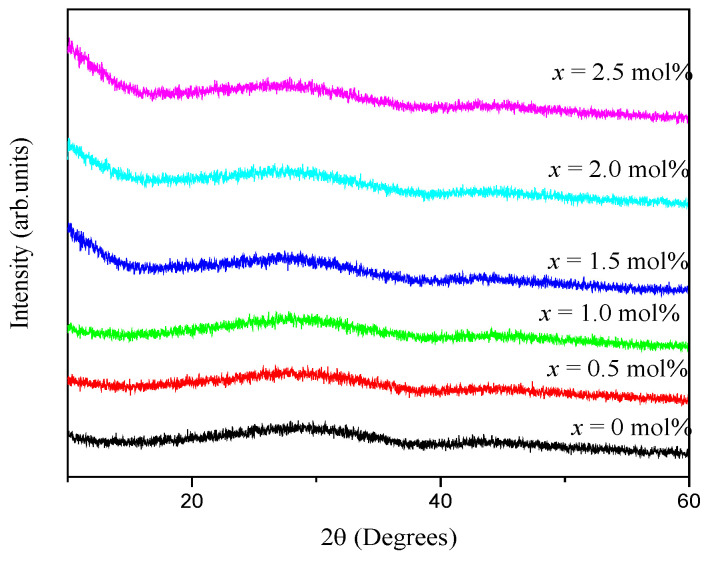
XRD patterns of (59.5–*x*) B_2_O_3_–20Na_2_O–20CaO–*x*V_2_O_5_–Er_2_O_3_–0.5AgCl (*x* = 0, 0.5, 1.0, 1.5, 2.0 and 2.5 mol%).

The XRD patterns of the (59.5–*x*) B_2_O_3_–20Na_2_O–20CaO–*x*V_2_O_5_–Er_2_O_3_–0.5AgCl (*x* = 0, 0.5, 1.0, 1.5, 2.0 and 2.5 mol%) glass samples are shown in Figure 1. All the samples showed two broad humps at approximately 20°–40° and 40°–60°. The presence of broad humps indicated the amorphous nature of all the glass samples.

**Figure 2:**
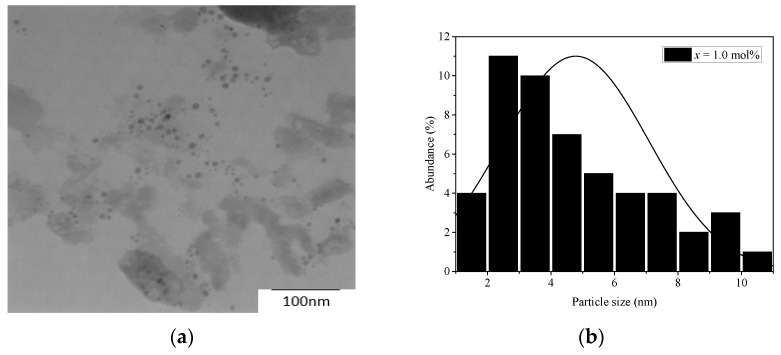
(**a**) TEM image with 100 nm magnification of glass with 1.0 mol% vanadium; (**b**) histogram of size distribution of metallic silver NPs.

Figure 2a shows a TEM image of a glass sample for *x* = 1.0 mol%. The TEM image shows a different size in non-spherical of dark spots. Figure 2b demonstrates the size of dark spots in the same glass with average size about 5 nm. The black spots in Figure 2a can be concluded as a silver NP.

**Figure 3:**
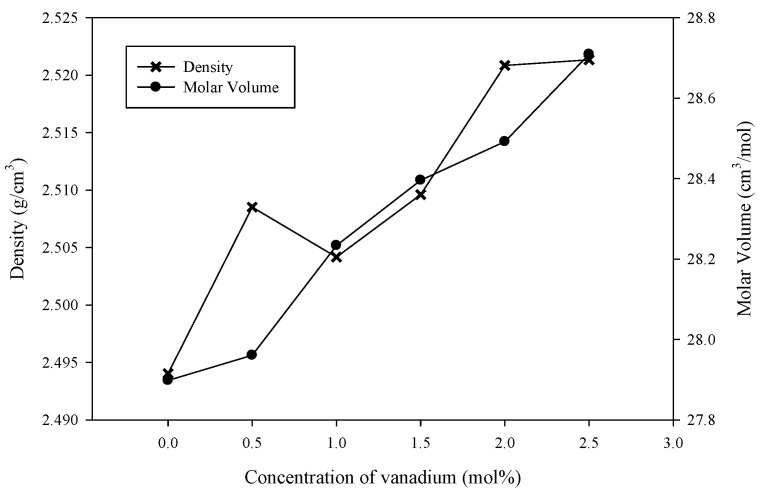
Density (*ρ*) and molar volume (*V_a_*) for (59.5–*x*) B_2_O_3_–20Na_2_O–20CaO–*x*V_2_O_5_–Er_2_O_3_–0.5AgCl (*x* = 0, 0.5, 1.0, 1.5, 2.0 and 2.5 mol%).

Table 1 provides the values of density, molar volume, and refractive index for (59.5–*x*) B_2_O_3_–20Na_2_O–20CaO–*x*V_2_O_5_–Er_2_O_3_–0.5AgCl (*x* = 0, 0.5, 1.0, 1.5, 2.0 and 2.5 mol%) glass samples. Figure 3 shows the variations in density and molar volume with the concentration of vanadium for the glass samples. The density of the samples displayed a nonlinear increment whilst the molar volume of the samples exhibited a monotonic increment. These sample patterns demonstrated harmonious concurrences relative to previous reports [5]. The density values were within 2.494 and 2.521 g cm^−3^ whilst the molar volumes were between 27.898 and 28.709 cm^3^ mol^−1^ with the addition of V_2_O_5_ into the glass samples. These density values were smaller than those of (60–*x*) B_2_O_3_–20Na_2_O–20CaO–*x*V_2_O_5_ (2.537–2.550 g cm^−3^), but the molar volume was greater than that of (60–*x*) B_2_O_3_–20Na_2_O–20CaO–*x*V_2_O_5_ (25.77–26.73 cm^3^ mol^−1^) [5].

Changes in molar mass and molar volume affect glass density. Moreover, density and molar volume usually show contradicting behaviors. In the present work, the density and molar volume displayed similar behavior, that is, both values increased with the addition of vanadium. The same behavior was reported for other borate glass systems [5,17].

The mass of B_2_O_3_ (M = 69.63 g mol^−1^) was lower than that of V_2_O_5_ (M = 181.88 g mol^−1^). Thus, the increase in density was due to the replacement of a lighter molecular component (B_2_O_3_) with a heavier molecular component (V_2_O_5_). Thus, the NBO increased with increasing V_2_O_5_ content.

The borate group consists of many B–O bonds, and vanadate groups contain various V–O bonds. The bonds in the borate group are shorter than the bonds in the vanadate group. According to [5], the bond lengths of BO_3_ and BO_4_ are 1.36 and 1.47 Å, respectively. By contrast, previous classical molecular (MD) stimulation research reported that the bond length of V^5+^–O (1.81–1.92 Å) was slightly longer than that of V^4+^–O (1.74–1.85 Å) [6]. Thus, the replacement of a short B–O bond length with a long V–O bond length can be expected to increase the molar volume and open the network structure of glass samples.

### 3.2. IR Spectra

Three active IR regions were observed in B_2_O_3_–V_2_O_5_ [18,19]. The first group of bands at around 500–750 cm^−1^ was due to the bending of the B–O–B linkages in the borate network. The second group of bands between 800 and 1200 cm^−1^ was due to the asymmetric vibration of BO_4_ units. The third group of bands at 1200–1450 cm^−1^ was due to the asymmetric stretching relaxation of the B–O band of the triagonal BO_3_ units. Other studies also reported the same results [17].

**Figure 4:**
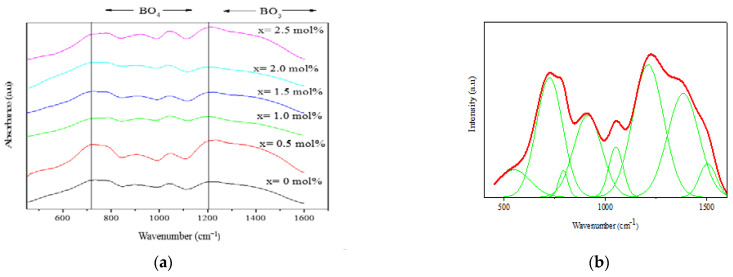
FTIR results of (**a**) (59.5–*x*) B_2_O_3_–20Na_2_O–20CaO–*x*V_2_O_5_–Er_2_O_3_–0.5AgCl (*x* = 0, 0.5, 1.0, 1.5, 2.0 and 2.5 mol%) and (**b**) deconvolution of 59B_2_O_3_–20Na_2_O–20CaO–0.5V_2_O_5_–Er_2_O_3_–0.5AgCl.

The IR absorption spectra for (59.5–*x*) B_2_O_3_–20Na_2_O–20CaO–*x*V_2_O_5_–Er_2_O_3_–0.5AgCl (*x* = 0, 0.5, 1.0, 1.5, 2.0 and 2.5 mol%) in the 400–1600 cm^−1^ region at room temperature were recorded, and the results are shown in Figure 4a. Figure 4b shows the deconvolution of the glass sample spectrum at *x* = 1.0 mol%. As shown in Figure 4, the vibrational modes of the borate network agreed with the results of previous research [5]. For the first region, the band between 1391 and 1407 cm^−1^ was attributed to the stretching vibration of the borate NBO bond which is BO_3_. The band correlated with the stretching vibration of B–O bonds in the group of BO_4_ units from tetraborate, pentaborate and tetraborate was located at about 927–1205 cm^−1^. Mohamed et al. [5] reported that the region at 990–1024 cm^−1^ overlapped with the vibration from the VO_5_ trigonal bipyramid unit of V = O. They assumed that the band of vibration for the isolated group B–O–V bridging bonds or V = O was located at 1000 cm^−1^. The region in 511–536 cm^−1^ was ascribed to the in-plane bending of B–O, and the IR band at around 740–751 cm^−1^ was ascribed to the B–O–B bending vibration of BO_4_ and BO_3_ [5].

In the evaluation of the impact of vanadium on the borate structure, the relative area of the BO_4_/V = O bands was normalized by the area of BO_4_ at *x* = 0 mol%. An addition of 0.5 mol% vanadium increased the relative area of the BO_3_ functional group. However, further addition of vanadium *x* > 0.5 mol% resulted in a decrease in the relative area of the BO_3_ functional group. The addition of 0.5 mol% V_2_O_5_ decreased the normalized plot of BO_4_/V = O, which then increased as the vanadium concentration increased in the glass samples. Increasing BO_4_/V = O and decreasing BO_3_ resulted in the formation of NBO, whereas increasing BO_3_ and decreasing in normalized BO_4_/V = O revealed an increasing BO [20]. In this study, the NBO increased when the concentration of vanadium *x* > 1.0 mol%.

### 3.3. UV–Vis Properties

**Figure 5:**
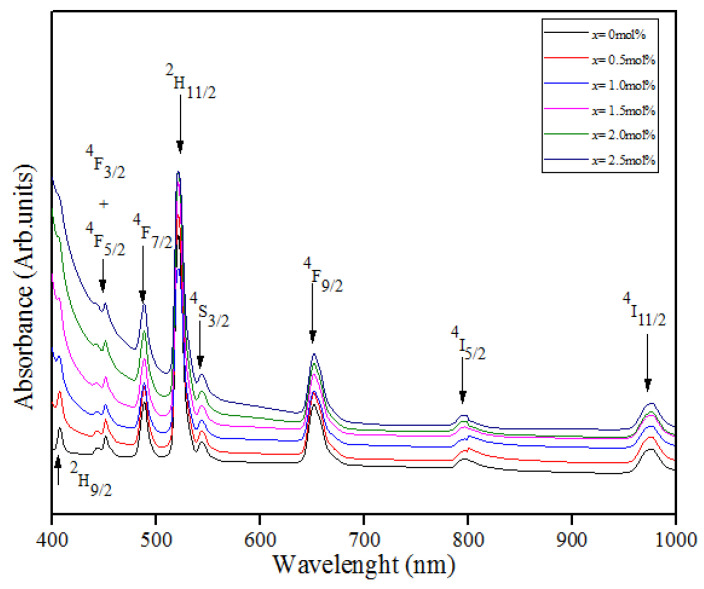
UV–Vis–NIR absorption spectra of (59.5–*x*) B_2_O_3_–20Na_2_O–20CaO–*x*V_2_O_5_–Er_2_O_3_–0.5AgCl (*x* = 0, 0.5, 1.0, 1.5, 2.0 and 2.5 mol%).

Figure 5 shows the absorption spectra for (59.5–*x*) B_2_O_3_–20Na_2_O–20CaO–*x*V_2_O_5_–Er_2_O_3_–0.5AgCl (x = 0, 0.5, 1.0, 1.5, 2.0 and 2.5 mol%). The absorption spectra contained six bands at 490, 520, 540, 660, 800 and 980 nm. In other findings, all the peaks were ascribed to the erbium absorption from the ground state ^4^I_15/2_ to the excited states ^4^F_7/2_, ^2^H_11/__2,_ ^4^S_3/2_, ^4^F_9/2_, ^4^1_9/2_ and ^4^1_11/2_ [7]. The comparison of the peaks showed that the transition of ^4^I_15/2_ → ^2^H_11/2_ with a wavelength of 520 nm presented the highest peak. No new band emerged with the addition of vanadium in the samples. This result was either due to the vanadyl ion not being observed in the recorded spectra or because it overlapped with the dominant erbium ion intensity band. The surface plasmon resonance (SPR) band contributed by silver NPs was also not observed. Previous studies reported that the SPR band is located at around 400–500 nm [21,22]. The SPR frequency depends on the refractive index (n ~ 2 for borate glass) and the dielectric function of silver [23].

### 3.4. Optical Properties

The optical properties of amorphous materials can be studied based on the electronic band structure and optical transition. If an electron in the valence band has enough energy, the electron rises across the band gap toward the conduction band. The energy required to cross the band gap is closely related to the optical energy band gap (E_opt_).

The optical absorption edge is used to investigate the electronic transition during absorption. The absorption coefficient (α) can be calculated at various wavelengths by using the Beer–Lambert Law [3].

**Figure 6:**
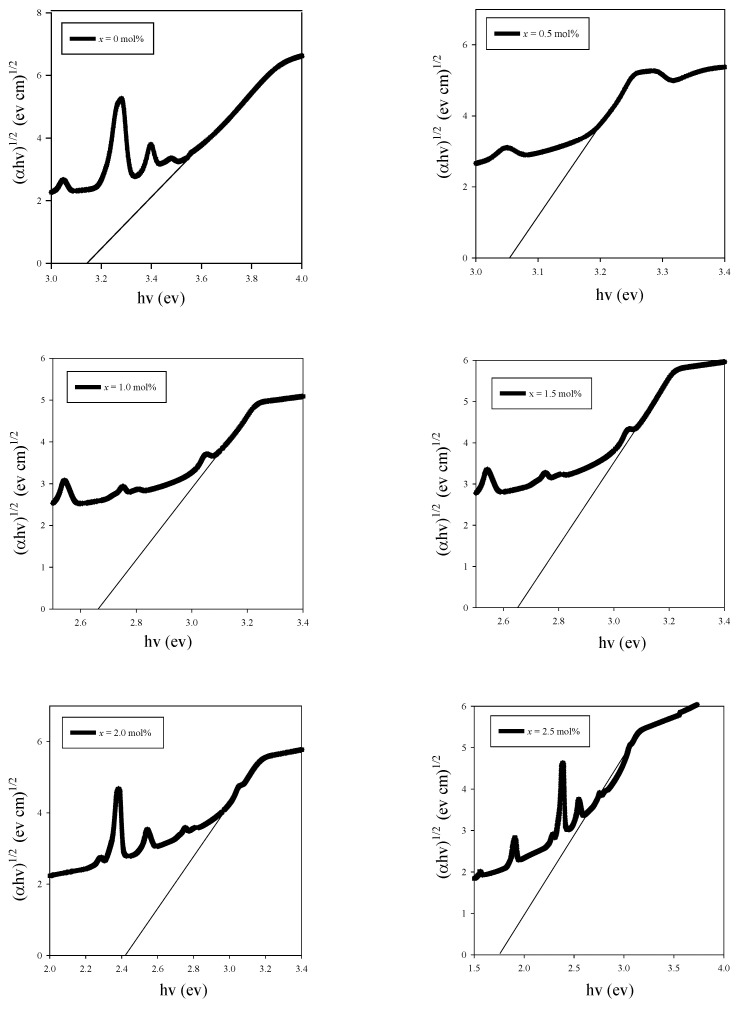
Straight line of curve at *x*-axis when (α*hv*)^1/2^ *=* 0. All the graph represents the typical extrapolation of Tauc’s plot of the glass samples.

A Tauc plot (Figure 6) was drawn according to the Davis and Mott relation with α [3]:

(2) $$\alpha \left(\omega \right)=\frac{A\;{\left(hv-E_{\mathrm{opt}}\right)}^{n}}{hv}$$

where *h* is the Planck constant, *v* is the photon frequency, *A* is a constant and n is a constant determining the types of transition. The n constant is equal to 2, 3, 1/2 or 1/3, which respectively denote indirect allowed, indirect forbidden, direct allowed and direct forbidden transition [9]. The value of n for the oxide glass is 2 [3]. In this study, the graph *hv* against (α*hv*)^2^ was plotted (Figure 6) and used to measure the optical band gap. The optical band gap is the intersection of the straight line of a curve at *x*-axis when (α*hv*)^2^ *=* 0 [24].

**Figure 7:**
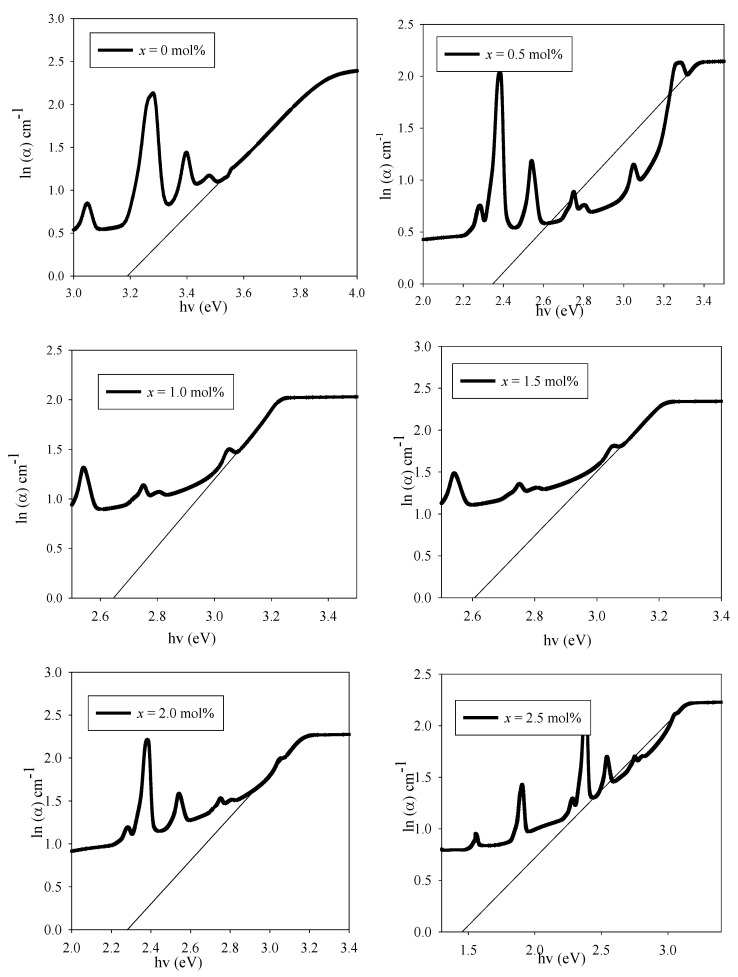
The graph of ln (α) against photon energy, hv (eV) of (59.5–*x*) B_2_O_3_–20Na_2_O–20CaO–*x*V_2_O_5_–Er_2_O_3_–0.5AgCl (*x* = 0, 0.5, 1.0, 1.5, 2.0 and 2.5 mol%). The graphs showed the determination of the Urbach energy for the glass samples.

The changes in band gap energy can be explained by the increase and decrease disorder in the material. Through Urbach’s equation, disorder can be calculated as [3]

(3) $$\alpha \left(v\right)=Cexp\left(\frac{hv}{Eu}\right)$$

where *α* is a constant and *Eu* is the Urbach energy. The graph of ln (*α*) versus *hv* is plotted in Figure 7. The reciprocal of the slope of the linear curve represents the Urbach energy [3]. Urbach energy depends on several factors, including temperature, average photon energy, induced disorder, static disorder, thermal vibration in the lattice and strong ionic bond.

**Figure 8:**
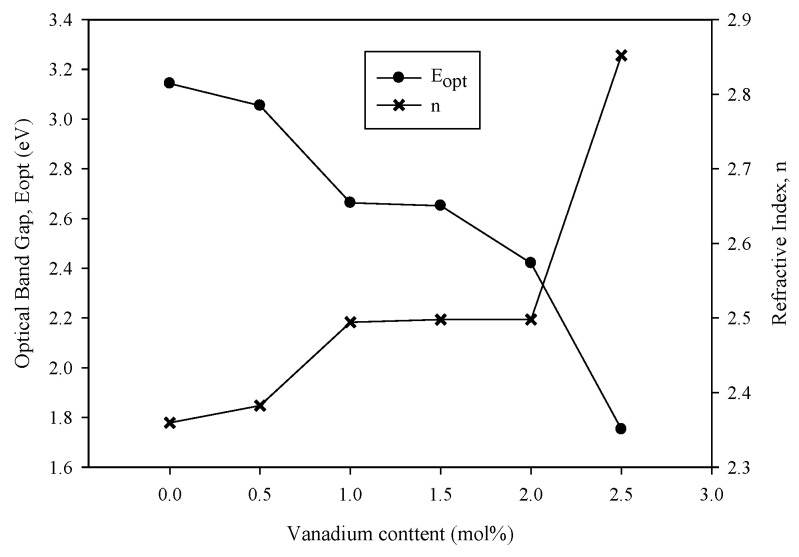
Optical band gap (E_opt_) and refractive index of (59.5–*x*) B_2_O_3_–20Na_2_O–20CaO –*x*V_2_O_5_–Er_2_O_3_–0.5AgCl (*x* = 0, 0.5, 1.0, 1.5, 2.0 and 2.5 mol%).

Table 2 shows the values of indirect energy band gap, Urbach energy and refractive index of all the samples. The energy band gap (E_opt_) values were in the range of 3.143–1.752 eV. E_opt_ decreased with an increase in vanadium concentration. As shown in Figure 8, the graphs of E_opt_ and *n* against V_2_O_5_ concentration showed contrasting behavior in which *n* increased when the vanadium concentration increased. This behavior was also found in previous research [3]. The *n* values were in the range of 2.360–2.852. In the studied glass samples, the reduction of the band gap with increased V_2_O_5_ was due to the structural evolution. For *x* < 1.0 mol%, the band gap decreased because of the increasing NBO in the borate triangular BO_3_ at a low concentration of vanadium. In contrast to that of BO, the creation of NBO opened the glass structure and resulted in an easier excitation of the electron because the electron showed a loose bond in NBO. The band gap being most likely constant for *x* = 1.5 mol% could be explained by the new role of V_2_O_5_ as a former oxide. Vanadium acts as a network modifier at a low concentration and as a network forming component at a high concentration [10]. The structural revolution that occurred by increasing the vanadium concentration caused the contrasting behavior of *n*.

**Figure 9:**
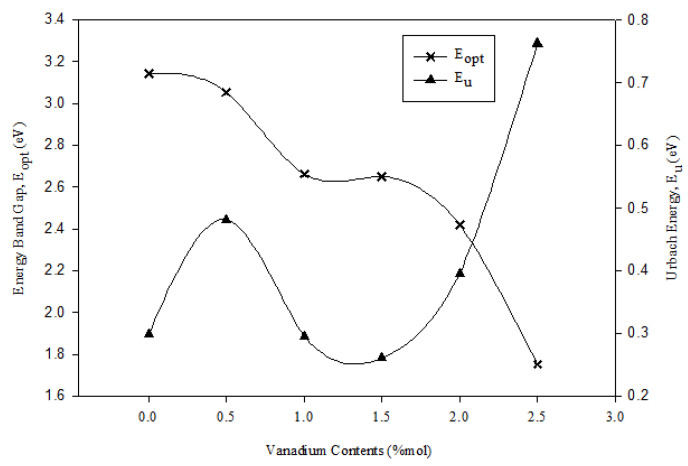
Optical band gap (E_opt_) and Urbach energy of (59.5–*x*) B_2_O_3_–20Na_2_O–20CaO –*x*V_2_O_5_–Er_2_O_3_–0.5AgCl (*x* = 0, 0.5, 1.0, 1.5, 2.0 and 2.5 mol%).

The E_U_ values of the glass increased sharply from 0.298 eV to 0.482 eV (*x* = 0 mol%–0.5 mol%), followed by a sharp decrease when 1.0 mol%–1.5 mol% of vanadium was added into the glass samples. The E_U_ values gradually increased to 0.762 (*x* = 2.5 mol%) with further increase in V_2_O_5_ content (Figure 9). The addition of vanadium *x* = 0.5 mol% increased the Urbach energy value, and this effect indicated the increased tendency of weak bonds to become defects. For *x* = 0.5 mol%, the concentration of the defect increased in the glass network with the increment of NBO. The Urbach energy value decreased for *x* = 1.0 and 1.5 mol%. This decrement suggested that the degree of disorder present in the glass decreased. However, for *x* > 1.5 mol%, the increase in Urbach energy reduced the energy band gap, whereas the decrease in Urbach energy increased the energy band gap.

### 3.5. Judd–Ofelt Analysis

Judd–Ofelt theory provides the information of transition behavior between 4f–4f electronic configuration and the calculation of oscillation strength, intensity parameter (Ω_2_, Ω_4_, Ω_6_), transition probabilities and branching ratio [25,26]. Judd–Ofelt theory is the best method to investigate and analyze the spectral properties of borate glass systems containing rare-earth ions (erbium ion). The absorption spectral data of all samples containing different concentrations of vanadium were used to calculate the Judd–Ofelt parameters. The precise integrated absorption cross-section measurement over the range of the wavelength and transition state of excitation is needed to analyze the Judd–Ofelt theory.

The area under the absorption band was used to determine the experimental oscillator strength. The experimental oscillator strength can be calculated via the following relation:

(4) $$f_{\exp}=\frac{2303\;mc^{2}}{\pi e^{2}N_{0}}\int^{\;}\varepsilon_{a}\left(v\right)dv$$

(5) $$=4.32\times 10^{-6}\;\int^{\;}\varepsilon \;\left(v\right)\;dv$$

where *m* is the electron mass, *c* is the velocity of light in vacuum, *N*_0_ is Avogadro’s number and ε(v)  is the molar extinction coefficient. The molar extinction coefficient was obtained from the measured absorbance of the samples calculated from the Beer–Lambert law as follows:

(6) $$\varepsilon_{a}\left(v\right)=\frac{\log_{0}\frac{I_{0}}{I_{t}}}{C_{RE}t}$$

where $\;C_{RE}$ is the concentration of the rare-earth ion (erbium) (mol/1000 cm^3^), *t* is the thickness of the samples in cm and $\log_{0}\frac{I_{0}}{I_{t}}$ is calculated from the absorbance from the wave number *v* (cm^−1^).

The estimation of theoretical oscillator strength for a transition from the ground state to an excitation state of erbium ion within the 4f configuration according to Judd–Ofelt theory is as follows:

(7) $$f_{cal}=\frac{8mcv\pi^{2}}{3h\left(2J+1\right)e^{2}}\frac{n^{2}+2}{9n}\;\left(\;e^{2}\sum_{\lambda =2,4,6}{Ω}_{\lambda }{\left|<aJ|U^{\left(\lambda \right)}|bJ\prime >\right|}^{2}\right)$$

where *v* is the wave number of the transition in cm^−1^, *h* is a Planck constant and *J* is the total angular momentum of the lowest state. The factor of (n^2^ + 2)/9n represents the electric field correction of Lorentz, and n is the refractive index of the samples. Ω*_λ_* is the Judd–Ofelt intensity parameter, where the *λ* values are 2, 4 and 6. $\left|\left|U^{\left(\lambda \right)}\right|\right|$ representing the double-reduced square matrix elements of the unit tensor operator of rank 𝜆 = 2, 4 and 6 was calculated using the intermediate coupling approximation method for the transition from the lowest state to the highest state. The reduced matrix elements ${Ω}_{\lambda }{\left|<aJ|U^{\left(\lambda \right)}|bJ\prime >\right|}^{2}$ were calculated following the work of Carnall et al. [27].

To evaluate the accuracy of the Judd–Ofelt parameter, this study identified the quality of fit by using the root mean square (rms) deviation relation given by

(8) $$rms={\left[\frac{\sum {\left(f_{calc}-f_{exp}\right)}^{2}}{\xi -3}\right]}^{1/2}$$

where ξ denotes the number of spectral bands analyzed (i.e., 3). The rms values indicated the quality of fit between the experimental and calculated oscillator strengths. These values also showed the accuracy of the Judd–Ofelt parameter.

Table 3 shows the calculated and experimental rms and oscillator strength of all the glass samples. The indirect data on the symmetry and bonding of rare-earth ions within the matrix were provided by the oscillator strength. The highest oscillator force attributed to the hypersensitive transition was reflected by the transition band at ^4^I_15/2_ → ^2^H_11/2_. Such hypersensitive transitions were sensitive to the changes in the local structure of the glass network. These hypersensitive transitions complied with ∆S = 0, |∆J| ≤ 2 and |∆L| ≤ 2 selection rules and reflected the interaction strength of erbium ions in the local network with the host glass. In the glass samples, the increase in vanadium content increased the oscillator strength of the hypersensitive transition. These changes revealed strong covalency with the presence of low symmetry around erbium ions. These values were found to equate to a higher oscillator strength than those of phosphate glass [28], boro-aluminosilicate glass [10] and tellurite glass [18,29]. In addition, the values of rms were in the range of 1–2 × 10. These considerably small rms values confirmed the accuracy of the data [30]. The rms values of all the glass samples conformed to those of a previous study [31].

Table 4 shows the values and trend of the Judd–Ofelt intensity parameters (Ω_2, 4, 6_) along with their spectroscopic properties (χ) for all the glass samples. The data of Judd–Ofelt parameters from the literature were compared with those of the current glass samples [17]. Glass composition determined the values of the Judd–Ofelt parameters. The increase in vanadium ion concentration from 0 mol% to 1.0 mol% decreased the Ω_2_ and Ω_6_ values from 3.19 × 10^−20^ to 2.43 × 10^−20^ and from 8.45 × 10^−21^ to 6.53 × 10^−21^, respectively. In addition, the trend of Ω_𝜆_ was found to be Ω_2_ > Ω_4_ > Ω_6_ for all the prepared glass samples. The samples with Ω_2_ and Ω_4_ had higher intensity parameters than the samples with Ω_6_ and were regarded as good glass hosts because of the high luminescence intensity ratio and high covalent bond between the erbium ions and local environment ligands [31,32]. The values of Ω_2_ and Ω_4_ were smaller for the borate glass containing erbium ions co-doped with vanadium than for the borate glass containing erbium ions only [17]. The parameters Ω_2_ and Ω_4_ were highly sensitive to the rare-earth ion’s local environment symmetry. The small values of Ω_2_ and Ω_4_ indicated the lower asymmetric nature of the local environment around the erbium ions in the glass system [21].

Ω_6_ contradicted Ω_2_ and Ω_4_, with Ω_6_ not being dependent on the local structure [33]; generally, the rigidity of glass is correlated with these parameters [31]. The glass without vanadium concentration (*x* = 0) was more rigid than the other glass samples (*x* = 0.5 mol%–2.5 mol%). As the value of Ω_6_ of the glass without vanadium was greater than those of the other samples with vanadium, the addition of vanadium ions from 0 mol% to 1.0 mol% led to the decrease of Ω_6_ values from 8.45 × 10^−21^ to 6.53 × 10^−21^. The result could be explained by the NBO which was created around the host matrix, where it caused high covalency and led to the production of high electron density for the ligand ions. 

The values of the Ω_4_ and Ω_6_ parameters were used to determine the spectroscopic quality factor (χ) [34]. χ defines the efficiency of laser transition. Therefore, it can be used to predict the stimulated emission of the laser. The values of χ for all the glass samples in this work were in the range of 1.70781–1.95143. These values were greater than those of erbium in tellurite glass systems [18]. The bigger the value of χ, the higher the efficiency of the laser transition because according to [35], the higher the value of χ, the more intense the laser transition. In this study, the glass with 1.0 mol% of vanadium was optically better than the other glass samples.

The values of Ω*_λ_* were used to calculate the radiative properties, such as the spontaneous emission rate (*A_R_*), branching ratio (*β_R_*) and lifetime of radiative transition (*τ_rad_*). The emission probabilities were called the Einstein coefficient for radiative transition *A_R_* (*aJ*, *bJ*’). The different transitions were calculated as.

(9) $$A_{R}\left(aJ,\;bJ\prime \right)=A_{ed}+A_{md}$$

where *A_ed_* is the electric dipole and *A_md_* is the magnetic dipole; both were calculated using Equations (9) and (10), respectively.

(10) $$\;\;A_{md}=\frac{64\;\pi^{4}v^{3}}{3h\left(2J+1\right))}\left(\chi_{md}S_{md}\right)$$

(11) $$\;\;A_{md}=\frac{64\;\pi^{4}v^{3}}{3h\left(2J+1\right))}\left(\chi_{md}S_{md}\right)$$

$\chi_{ed}$ and $\chi_{md}$ denote the local-field correction for the electric dipole and magnetic dipole transition, respectively. Both were obtained using the following relations:

(12) $$\chi_{ed}=\frac{n{\left(n^{2}+2\right)}^{2}{}^{}}{9}$$

(13) $$\chi_{md}=n^{3}$$

The line strengths of the magnetic dipole and electric dipole transition are represented by $S_{md}$ and $S_{ed}$, respectively. Both were calculated by the following relations:

(14) $$S_{md}\left(aJ,bJ\prime \right)=\frac{e^{2}}{4m^{2}c^{2}}{\left|<aJ|L+2S|bJ\prime >\right|}^{2}$$

(15) $$S_{ed}\left(aJ,bJ\prime \right)=e^{2}\underset{\lambda =2,4,6}{\sum }{Ω}_{\lambda }{\left|<aJ|U^{\left(\lambda \right)}|bJ\prime >\right|}^{2}$$

As shown in Table 5, the ^4^I_15/2 →_ ^2^H_11/2_ and ^4^I_15/2 →_ ^4^F_7/2_ transitions had high *A_R_* values. In addition, ^2^H_11/2_ and ^4^F_7/2_ showed increased *A_R_* values when the concentration of vanadium in the glass samples increased. This result indicated that the transitions of ^2^H_11/2_ and ^4^F_7/2_ benefitted the green and blue emissions, which are suitable for lasers [17].

Radiative lifetime carries important data for laser and optical amplifiers. The radiative lifetime (*τ_rad_*) of the prepared glass was a reciprocal of the total transition probabilities of emission state $\sum_{bJ\prime }A\left(aJ,bJ^{\prime }\right)$ (sum of transition probabilities of all the transitions from the highest state to the various lower states).

(16) $$\tau_{rad\;}=\frac{1}{\sum_{bJ\prime }A\left(aJ,bJ^{\prime }\right)}$$

The emission branching ratio is given as

(17) $$\beta_{R}\left(aJ,\;aJ\prime \right)=\frac{A_{R}\left(aJ,\;bJ\prime \right)}{\sum_{bJ\prime }A_{R}\left(aJ,\;bJ\prime \right)}$$

The obtained values of τ and $\beta_{R}$ are listed in Table 5. The probability of simulated emission acquisition can be determined by the values of the branching ratio for a specific transition [21]. The branching ratio for the transitions of ^4^I_15/2 →_ ^2^H_11/2_ and ^4^I_15/2 →_ ^4^F_7/2_ was 99%, and the value of the radiative ratio was in the range of 0.007–0.005. The value of the present glass was shorter than those of other glass systems. The short radiative lifetime of this transition was beneficial for the control of the strong emission of erbium ions within the prepared glass system and the suppression of the nonradiative process.

### 3.6. Photoluminescence

**Figure 10:**
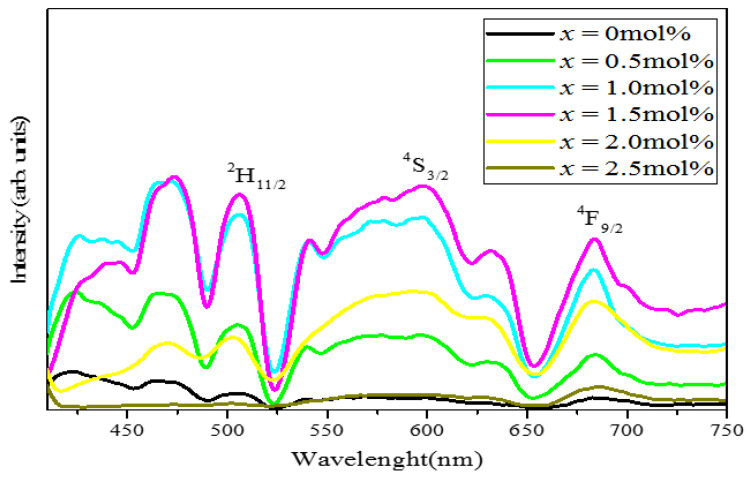
Emission spectra of all glass samples with excitation wavelength at 800 nm.

Figure 10 reveals the PL emission spectra of the (59.5–*x*) B_2_O_3_–20Na_2_O–20CaO–*x*V_2_O_5_–Er_2_O_3_–0.5AgCl (*x* = 0, 0.5, 1.0, 1.5, 2.0 and 2.5 mol%) glass samples in the wavelength ranging from 400 nm to 800 nm. The excitation wavelength was 800 nm. The emission spectra of the Er^3+^ ions exhibited three dominant peaks at 516, 580 and 673 nm. These peaks were ascribed to ^2^H_11/2_–^4^I_15/2_, ^4^S_3/2_–^4^I_15/2_ and ^4^F_15/2_–^4^I_15/2_. The bands at 516, 580 and 673 nm were due to the stark splitting effects resulting from the low symmetry of the local environment around the erbium ions [36]. The intensity increased when the concentration of V_2_O_5_ increased from 0 mol% to 1.5 mol%. However, when the concentration of vanadium exceeded 1.5 mol%, the intensity decreased. Thus, the decrement was due to concentration quenching [37,38]. At a high amount of vanadium, the excess vanadium ions produce structural defects that cause nonradiative recombination. Herein, the transition from the excited state to the visible wavelength was related to the emission of peaks at 516, 580 and 673 nm [36].

## 4. Conclusions

The effects of V_2_O_5_ substitution on the structural and optical properties of (59.5–*x*) B_2_O_3_–20Na_2_O–20CaO–*x*V_2_O_5_–Er_2_O_3_–0.5AgCl (*x* = 0, 0.5, 1.0, 1.5, 2.0 and 2.5 mol%) glasses were investigated. The glass samples were successfully prepared via the melt-quenching method. The structural properties of the samples based on XRD indicated their amorphous nature without any crystalline phase following the addition of vanadium. Meanwhile, the FTIR revealed the structural units in the glass network. Specifically, the FTIR confirmed the presence of B-O-B stretching in the borate network, bending vibration from the O–V–O of the VO_4_ tetrahedral group, vibration of the B–O–V bridging bond and stretching vibration of the B–O bond belonging to BO_3_. The addition of at least 1.0 mol% vanadium created additional NBO in the glass structure. The Vis–NIR spectra exhibited eight absorption bands at 490, 520, 540, 660, 800 and 980 nm. All the peaks denoted the erbium absorption from the ground state ^4^I_15/2_ to the excited states ^4^F_7/2_, ^2^H_11/2,_ ^4^S_3/2_, ^4^F_9/2_, ^4^1_9/2_ and ^4^1_11/2_. The most intense peak was centered at 540 nm and was called the hypersensitive transition. The SPR band and vanadium band were not observed in the recorded spectra because of the low concentration of silver NPs and the dominance of the erbium band. The increase in vanadium was found to reduce the band gap energy and increase the refractive index and Urbach energy from 2.360 to 2.852 and from 0.298 eV to 0.762 eV, respectively. According to the Judd–Ofelt principle, the spectroscopic parameter was calculated. The calculation involved the Judd–Ofelt parameters, quality factors, radiative lifetime and branching ratio. The Judd–Ofelt parameters were revealed to follow the trend of Ω_2_ > Ω_4_ > Ω_6_. The PL spectra exhibited three bands at 516, 580 and 673 nm, which were represented as ^2^H_11/2_–^4^I_15/2_, ^4^S_3/2_–^4^I_15/2_ and ^4^F_15/2_–^4^I_15/2_, respectively. The obtained Judd–Ofelt parameters and the PL results showed that the glass samples with 1.0 mol% of vanadium were useful in green laser application because of their high lifetime value, branching ratio and strong spectral intensity relative to other samples.

## Figures and Tables

**Table 1:** Values of density (*ρ*) and molar volume (*V_a_*) for (59.5–*x*) B_2_O_3_–20Na_2_O–20CaO–*x*V_2_O_5_–Er_2_O_3_–0.5AgCl (*x* = 0, 0.5, 1.0, 1.5, 2.0 and 2.5 mol%).

Samples (mol%)	Density (g cm^−3^)	Molar Volume (cm^3^ mol^−1^)
*x* = 0	2.494	27.898
*x* = 0.5	2.507	27.960
*x* = 1.0	2.504	28.233
*x* = 1.5	2.509	28.395
*x* = 2.0	2.520	28.492
*x* = 2.5	2.521	28.709

**Table 2:** Indirect optical energy band gap (E_opt_), Urbach energy (E_U_) and refractive index (*n*) of (59.5–*x*) B_2_O_3_–20Na_2_O–20CaO–*x*V_2_O_5_–Er_2_O_3_–0.5AgCl (*x* = 0, 0.5, 1.0, 1.5, 2.0 and 2.5 mol%).

*x* (mol%)	E_opt_ (eV)	E_U_ (eV)	*n*
0	3.143	0.298	2.360
0.5	3.054	0.482	2.383
1.0	2.663	0.296	2.494
1.5	2.651	0.261	2.498
2.0	2.421	0.395	2.498
2.5	1.752	0.762	2.852

**Table 3:** Calculated oscillator strength (f_cal_, 10^−6^), experimental oscillator strength (f_exp_, 10^−6^) and rms of erbium absorption transition from the ground state ^4^I_15/2_ to the excited state.

Transition ^4^I_15/2_→	Glass Samples											
	*x* = 0 mol%		*x* = 0.5 mol%		*x* = 1.0 mol%		*x* = 1.5 mol%		*x* = 2.0 mol%		*x* = 2.5 mol%	
	fcal	fexp	fcal	fexp	fcal	fexp	fcal	fexp	fcal	fexp	fcal	fexp
^4^I_11/2_	7.95	6.99	7.65	7.32	6.83	6.81	8.05	7.32	8.12	7.93	7.78	7.7
^4^I_9/2_	5.95	4.86	6.31	6.02	5.81	6.24	6.09	5.1	6.38	4.96	6.13	4.63
^4^F_9/2_	3.27	3.27	3.36	3.35	3.08	3.05	3.3	3.3	3.42	3.46	3.29	3.35
^4^S_3/2_	6.28	4.92	5.96	4.29	5.41	4.02	6.08	5.22	6.23	4.55	6.06	4.41
^2^H_11/2_	1.03	1.03	1.05	1.05	9.07	9.07	1.19	1.19	1.15	1.15	1.06	1.06
^4^F_7/2_	2.78	2.95	2.73	2.85	2.49	2.58	2.73	2.85	2.82	2.87	2.73	2.76
rms	1.53		1.22		1.03		1.10		1.33		2.86	

**Table 4:** Judd–Ofelt intensity parameters and spectroscopic quality factors for all prepared glass samples.

Glass Samples	Ω_2_ cm^2^	Ω_4_ cm^2^	Ω_6_ cm^2^	Trends of Ω*_λ_*	χ = Ω_4_/Ω_6_	Ref.
*x* = 0 mol%	3.19 × 10^−20^	1.44 × 10^−20^	8.45 × 10^−21^	Ω_2_ > Ω_4_ > Ω_6_	1.708	This work
*x* = 0.5 mol%	3.16 × 10^−20^	1.51 × 10^−20^	7.87 × 10^−21^	Ω_2_ > Ω_4_ > Ω_6_	1.924	This work
*x* = 1.0 mol%	2.43 × 10^−20^	1.27 × 10^−20^	6.53 × 10^−21^	Ω_2_ > Ω_4_ > Ω_6_	1.951	This work
*x* = 1.5 mol%	3.38 × 10^−20^	1.32 × 10^−20^	7.30 × 10^−21^	Ω_2_ > Ω_4_ > Ω_6_	1.812	This work
*x* = 2.0 mol%	3.21 × 10^−20^	1.39 × 10^−20^	7.49 × 10^−21^	Ω_2_ > Ω_4_ > Ω_6_	1.850	This work
*x* = 2.5 mol%	2.22 × 10^−20^	1.00 × 10^−20^	5.50 × 10^−21^	Ω_2_ > Ω_4_ > Ω_6_	1.826	This work
LiBEr5	4.39 × 10^−20^	3.22 × 10^−20^	5.50 × 10^−21^	Ω_2_ > Ω_4_ > Ω_6_	5.85	[17]
BLNEr	3.35 × 10^−20^	1.34 × 10^−20^	7.89 × 10^−21^	Ω_2_ > Ω_4_ > Ω_6_	1.69	[22]

**Table 5:** Values of *A_R_* (s^−1^), *β_R_* (%) and τ (ms) of all prepared glass samples.

Trans.	Para.	*x* = 0 mol%	*x* = 0.5 mol%	*x* = 1.0 mol%	*x* = 1.5 mol%	*x* = 2.0 mol%	*x* = 2.5 mol%
^4^I_15/2 →_^4^I_11/2_	*A* (s^−1^)	350.450	345.077	342.610	399.333	402.134	510.599
	*β_R_* (%)	88.284	87.946	86.740	87.979	88.033	86.711
	τ (ms)	0.285	0.290	0.292	0.250	0.249	0.196
^4^I_15/2 →_ ^4^I_9/2_	*A* (s^−1^)	388.305	417.475	421.780	446.074	466.749	585.557
	*β_R_* (%)	89.351	89.291	89.174	89.322	89.362	89.197
	τ (ms)	0.258	0.240	0.237	0.224	0.214	0.171
^4^I_15/2 →_ ^4^F_9/2_	*A* (s^−1^)	3528.567	3702.558	3723.815	3982.301	4127.788	5193.279
	*β_R_* (%)	80.950	80.958	80.897	80.927	80.874	80.792
	τ (ms)	0.028	0.027	0.027	0.025	0.024	0.019
^4^I_15/2 →_ ^4^S_3/2_	*A* (s^−1^)	1104.706	1069.927	1066.042	1198.596	1229.809	1561.382
	*β_R_* (%)	71.196	71.162	71.074	71.145	71.160	71.060
	τ (ms)	0.091	0.093	0.094	0.083	0.081	0.064
^4^I_15/2 →_ ^2^H_11/2_	*A* (s^−1^)	14239.160	14798.610	13983.960	18283.850	17738.210	21451.450
	*β_R_* (%)	99.182	99.190	99.017	99.245	99.221	99.041
	τ (ms)	0.007	0.007	0.007	0.005	0.006	0.005
^4^I_15/2 →_ ^4^F_7/2_	*A* (s^−1^)	4326.441	4339.731	4338.381	4773.069	4928.462	6225.356
	*β_R_* (%)	99.924	99.922	99.911	99.918	99.921	99.907
	τ (ms)	0.023	0.023	0.023	0.021	0.020	0.016

## Data Availability

The data presented in this study are available on request from the corresponding author.
